# P-SILI is not justification for intubation of COVID-19 patients

**DOI:** 10.1186/s13613-020-00724-1

**Published:** 2020-08-03

**Authors:** Martin J. Tobin, Franco Laghi, Amal Jubran

**Affiliations:** Division of Pulmonary and Critical Care Medicine, Hines Veterans Affairs Hospital and Loyola University of Chicago Stritch School of Medicine, Hines, IL 60141 USA

Dear Editor,

We thank Dr. Gattinoni and colleagues for their interest in our article and their thought-provoking comments [1, 2].

They are correct in observing we quoted opinion articles: three were by Gattinoni et al. We will not point out all instances where Gattinoni et al. misquoted our article, but two need to be addressed. One, they claim we communicated “very few persons require intubation”—we never said that. Two, they state “Tobin et al.…use this to suggest that invasive ventilation is fatal.” On the contrary, we wrote “Mechanical ventilation is lifesaving in severe respiratory failure, and few medical therapies equal its power” [2].

In reference to experimental evidence supporting the existence of patient self-induced lung injury (P-SILI), Gattinoni and colleagues note that “Barach exploited spontaneous breathing to induce experimental lung oedema” [1]. On the contrary, Barach et al. are explicit in stating that they were “unable to confirm…that a pathologically elevated negative pressure was responsible for the occurrence of pulmonary edema” (page 770). It is true that pulmonary edema can result from large pleural pressure swings, such as consequent to upper airway obstruction. Patients with acute severe asthma develop large pleural pressure swings, yet autopsy studies in patients dying because of status asthmaticus are remarkable for the absence of pulmonary edema [3].

We are unsure what Gattinoni et al. [1] mean when they claim we cited the study of Mascheroni et al. misleadingly. In addition to previously highlighted problems, we add that 31% of hyperventilating sheep died without life-threatening hypoxemia, that surfactant properties in afflicted sheep were equivalent to control animals, the absence of a control group of sheep ventilated with ventilator settings that mimicked the breathing pattern of the non-intubated sheep, and *en passant* dismissal of neurogenic pulmonary edema. These flaws need to be underscored about a study regarded as an experimental foundation for the existence of P-SILI.

Gattinoni et al. [1] claim that the study by Tonelli et al. supports the existence of P-SILI. It does not. Tonelli et al. did record large swings in esophageal pressure (ΔPes), but did not document regional lung damage. If inspiratory efforts were causing P-SILI, one would expect a decrease in tidal volume-to-transpulmonary pressure swing ratio (*V*_T_/ΔP_L_)—a surrogate of lung compliance. *V*_*T*_/ΔP_L_ remained constant across 24 h of noninvasive ventilation (see Supplement: Figure E2, panel C in Tonelli et al). Worsening chest radiographs at 24 h cannot be linked mechanistically to P-SILI (or failure of noninvasive ventilation) because the radiographs were taken following intubation (to which a radiologist cannot be blinded).

Gattinoni and colleagues [1] note that frequency-to-tidal volume (f/*V*_*T*_) is expressed with a threshold value. The f/*V*_*T*_ threshold was derived by first analyzing a training data set, and then accuracy of that f/*V*_*T*_ threshold was tested prospectively in a subsequent validation data set [4]. We used the same approach in our Pes weaning study [5]. This rigorous approach differs fundamentally from picking ΔPes of 15 cmH_2_O based on theoretical rationalization without any experimental testing.

Gattinoni and colleagues’ recommendations regarding intubation in COVID-19 patients were explicit, without caveats: “intubation should be prioritized”, and when ΔPes increases above 15 cmH_2_O, “intubation should be performed as soon as possible” [2]. We are relieved they no longer recommend early intubation. They now “advocate avoiding delayed intubation”—but delayed intubation is a diagnosis that can be made only in hindsight.

We are pleased that Gattinoni et al. [1] have reversed their advice on weaning of COVID-19 patients and no longer recommend that “weaning should be undertaken cautiously” [2]. It is true that the rate of intubation and mortality in COVID-19 patients exhibits a broad range. All the more reason to avoid issuing explicit directions based on binary alliterative (H, L) ARDS phenotypes—as yet untested.

To help readers better understand the importance of P-SILI in influencing intubation and ventilator weaning in COVID-19 patients, we hope that Gattinoni and colleagues will answer the following questions:

- What *experimentum crucis* has been undertaken in humans to demonstrate that vigorous inspiratory efforts cause P-SILI?
- What calculus can they provide for the tradeoff between decades of documented complications consequent to intubation and mechanical ventilation versus the hypothesized existence of P-SILI?

We are not saying that P-SILI is an uninteresting hypothesis. We are concerned about recommendations for intubation and ventilator weaning during the COVID-19 pandemic based on an untested hypothetical entity.

## Data Availability

Not applicable.

## Abbreviations

COVID-19: Coronavirus Disease 2019
f/*V*_*T*_: Frequency-to-tidal volume
Pes: Esophageal pressure
P-SILI: Patient self-induced lung injury
*V*_*T*_/ΔP_L_: Tidal volume-to-transpulmonary pressure swing ratio
ΔPes: Tidal swings in esophageal pressure
